# Nitrogen addition substantially affects plant phenology in terrestrial ecosystems: a meta-analysis

**DOI:** 10.3389/fpls.2025.1632357

**Published:** 2025-08-19

**Authors:** Yaqi He, Tenglong Zhou, Jianing Mao, Mengke Li, Ruomin Sun, Chang Liu, Wen Li, Lei Ma, Shenglei Fu

**Affiliations:** ^1^ College of Geographical Sciences, Faculty of Geographic Science and Engineering, Henan University, Zhengzhou, China; ^2^ Dabieshan National Observation and Research Field Station of Forest Ecosystem at Henan, Kaifeng, China; ^3^ Key Laboratory of Geospatial Technology for Middle and Lower Yellow River Regions (Henan University), Ministry of Education, Kaifeng, China; ^4^ Xinyang Academy of Ecological Research, Henan University, Xinyang, China

**Keywords:** phenological delay, nitrogen deposition, ecosystem comparison, meta-analysis, climatic response

## Abstract

**Introduction:**

Phenology is a sensitive biological indicator of climate change. Increasing nitrogen (N) deposition has amplified phenological shifts, making their study across terrestrial ecosystems crucial for understanding global change responses. While existing research focuses on single ecosystems, comparative analyses are lacking.

**Methods:**

Our meta-analysis of 125 species across different ecosystems examined the effects of nitrogen addition on various phenological stages.

**Results:**

The results showed that nitrogen addition advanced flowering by 0.18 days but delayed budding (4.15d), leaf fall (4.95d), fruiting (0.21d), leaf discoloration (1.5d), maturity (3.3d), senescence (4d), and xylem growth (8.56d). In summary, nitrogen addition remarkably affects terrestrial ecosystems by delaying most phenological stages of plants. Additionally, some climatic factors also significantly influence phenological stages. A positive correlation exists between temperature elevation and the advancement of key phenological stages (e.g., flowering) in forest ecosystems, while temperature, precipitation, and nitrogen addition had little effect on the phenology of grassland and farmland plants.

**Discussion:**

This is because different ecosystems have different functions, resource allocation, and climate adaptation strategies, resulting in different responses to different environmental factors. Thus, future research should focus on how global changes affect the phenology of plants in different ecosystems rather than in single ecosystems.

## 1 Introduction

Phenology is a natural phenomenon characterized by periodic changes influenced by environmental conditions that occurs on scales ranging from individual organisms to entire ecosystems, thus representing a dominant aspect of ecology (Fu et al., 2022). As a specialized subdiscipline, plant phenology systematically investigates the timing of key life cycle events (e.g., germination, flowering, fruiting, and senescence) and their interactions with environmental variables, thereby serving as a critical bioindicator of terrestrial ecosystem dynamics (Caparros-Santiago et al., 2021). Phenological changes have long been considered sensitive indicators of climate change (Zhao et al., 2013). The response and feedback of vegetation phenology to climate change directly or indirectly affect a range of biogeochemical and biophysical processes, including carbon source–sink conversions and ecosystem water balance within terrestrial ecosystems. Human activities, such as fertilizer application and industrial development, have exponentially increased nitrogen input to the Earth’s land surface (Li et al., 2025). Recent statistical data indicate that human activities have increased the global emissions of reactive nitrogen by more than three times the natural output (WallisDeVries and Bobbink, 2017). Nitrogen enrichment has been shown to decrease the quality of surface and groundwater, acidify soils, and eutrophicate water bodies (Liu et al., 2011). It affects carbon sequestration, alters plant species composition, affects ecosystem structure and function, and limits primary and secondary community production (Yang et al., 2024). Therefore, the intensification of global nitrogen deposition is considered a significant driving factor affecting the structure and function of terrestrial ecosystems (Stevens et al., 2022) and, thus, has garnered widespread attention from ecologists. Consequently, the effect of nitrogen deposition on terrestrial ecosystems is currently a research hotspot (Smith et al., 2012; Xia and Wan, 2013).

Global change is driven by multiple factors that may interact synergistically or antagonistically, thereby influencing plant phenology and increasing the complexity of phenological predictions under future climate scenarios. The combined effects of these drivers can be either purely additive or reflect nonlinear interactions among them (Zhou et al., 2023). Specifically, nitrogen deposition alters the transition from vegetative growth to reproductive phases, significantly modifying plant phenological patterns (Gao et al., 2020). Furthermore, temperature and nitrogen availability jointly regulate plant nutrient cycling, with global warming and enhanced nitrogen deposition exhibiting interactive effects on plant growth and developmental processes (Elrys et al., 2024). These effects are particularly evident in shifts in plant phenology: studies show that nitrogen addition delays the phenological period of different plant types in grassland ecosystems to varying degrees. Moreover, it also affects the phenology of some forest plants and consistently delays phenological phases in both farmland and grassland ecosystems.

Grassland community experiments showed that plant phenology indicated shifts in life history strategies and revealed that nitrogen addition slightly advanced the flowering period of forbs while significantly delaying the flowering of grasses, without extending the flowering duration of grasses (Cleland et al., 2007; Yang et al., 2020). A study conducted in a grassland demonstrated that nitrogen addition had no impact on flowering, fruiting, or reproductive growth timing at both the species and community levels (He et al., 2016). Research on northern coniferous forest communities in eastern Canada showed that canopy nitrogen addition and soil warming led to earlier germination and faster bud development in conifer seedlings (Marty et al., 2020). Another study showed that, in forest ecosystem, nitrogen addition delayed the autumn phenology of Chinese Larix (Wang et al., 2022). In contrast, experiments in temperate desert ecosystems indicated that both nitrogen and water advanced the flowering and fruiting times of spring ephemerals (Huang et al., 2018). In alpine ecosystems, plants are strong indicators of phenological responses. In subalpine meadows, nitrogen addition significantly delays the onset of reproductive phenology in early-flowering sedges but advances the end of reproductive phenology in late-flowering sedges (Yang et al., 2023a). An examination of the effect of nitrogen addition on crop phenology concluded that an appropriate amount of nitrogen addition could advance the heading stage of crops and improve crop yield (Amanullah et al., 2009). In conclusion, although plant phenology changes under nitrogen addition, the phenological responses to nitrogen addition have not been as clearly illustrated as the responses to other environmental factors. Most existing studies have focused on individual ecosystems, limiting a comprehensive understanding of how nitrogen addition influences plant phenology across diverse terrestrial ecosystems. Thus, a broader-scale assessment of phenological stage-specific responses to nitrogen addition is needed. Research indicates that nitrogen addition and reduced precipitation exert antagonistic effects, leading to divergent impacts on plant phenology (Reich et al., 2020). However, the effects of nitrogen deposition on plant phenology to temperature and precipitation as well as phenological period of plants have not been clarified.

This study investigated the effects of nitrogen addition or deposition on the phenology of different terrestrial ecosystems by compiling a dataset of 125 species and conducting a comprehensive meta-analysis. The main questions were as follows: 1) How do the phenological stages of different ecosystems respond to nitrogen addition? 2) How do different ecosystems respond to nitrogen addition? 3) How do environmental factors affect the phenological phases of different species in the context of nitrogen addition?

## 2 Materials and methods

### 2.1 Literature search

Relevant literature published between 2000 and 2024 on the effect of nitrogen addition on plant phenology was examined on May 25, 2024, using online databases, including Google Scholar, ISI Web of Science, and CNKI. Our literature search included journals, conferences, reports, and book chapters. The search terms used were as follows: (nitrogen OR urea OR fertilizer * OR nitrogen fertilizer * OR nutrient *) AND (budding OR phenology * OR reproduction * OR flowering * OR senescence * OR fruiting * OR maturation * OR growing season * OR duration) AND (impact * OR response * OR increase * OR decrease * OR change *) AND (terrestrial ecosystems * OR forests * OR grasslands * OR farmlands *). The retrieved papers were then reviewed to identify previous studies.

### 2.2 Literature inclusion and exclusion criteria

The collected literature needed to satisfy the following criteria to be included in this study: 1) Studies conducted before 2000 were excluded due to substantial shifts in the chemical composition of global nitrogen deposition and intensified climate change interactions around the turn of the century. Data from pre-2000 studies would exhibit significant discrepancies compared to recent datasets, thereby compromising the validity of the analysis. Additionally, research prior to 2000 predominantly relied on manual ground-based observations, which introduced higher subjectivity and lower temporal resolution, further widening the methodological gap with contemporary studies and undermining the accuracy of analytical outcomes. 2) The study must employ analysis of variance or linear mixed model methodologies and provide data on sample size, mean, and standard deviation (SD) or error (SE) to ensure the accuracy of the experimental data. 3) The experiment must contain at least one dataset (treatment and control) within the same spatial and temporal scales. Articles with experimental data for the treatment without a control group were excluded because the absence of data from a control group cannot reflect how plant phenology responds to nitrogen addition. 4) Nitrogen addition must be performed through proactive nitrogen augmentation experiments; articles without nitrogen augmentation measures were excluded. 5) Studies in which experimental data were obtained from NDVI data were excluded because remote sensing data differs greatly from field data and do not provide a very accurate picture of how nitrogen addition affects plant phenology. 6) The experimental data must be sourced from field experiments, excluding data derived from theoretical models, to ensure that the data are representative and accurate. 7) The study must have collected data from at least one phenological phase. 8) Given that only one available study was identified for each desert and tundra ecosystem, the sample size is statistically insufficient to support reliable analysis. In accordance with the systematic review methodology guidelines (specifically Item 12 of the PRISMA guidelines) and standard meta-analytic protocols (Borenstein, 2023), studies from these two ecosystem types were temporarily excluded during heterogeneity testing and effect size pooling. This approach adheres to the minimum subgroup sample requirements for meta-analysis while ensuring the validity of comparisons among major ecosystem types. Overall, 51 papers on nitrogen addition or deposition were finally selected through screening.

### 2.3 Data extraction

Data were extracted from the literature, with sources including text, tables, and figures. To extract data from figures, Web Plot Digitizer software (https://apps.automeris.io/wpd4/) (Burda et al., 2017) was used, and the data were organized and analyzed in terms of the mean, SD, SE, and sample size (N) for the treatment and control groups. All data were raw data. For studies that did not report the SD or SE, we propose a novel method to address missing SDs by estimating a weighted average coefficient of variation (CV) from studies in datasets that report SDs. Use the impute_SD() function of the metagear package in R to imputate the missing SD value (Nakagawa et al., 2023). The SE values reported in the literature were converted using Equation 1.


(1) $$SD=\text{S}E\sqrt{N}$$

**Figure 1:**
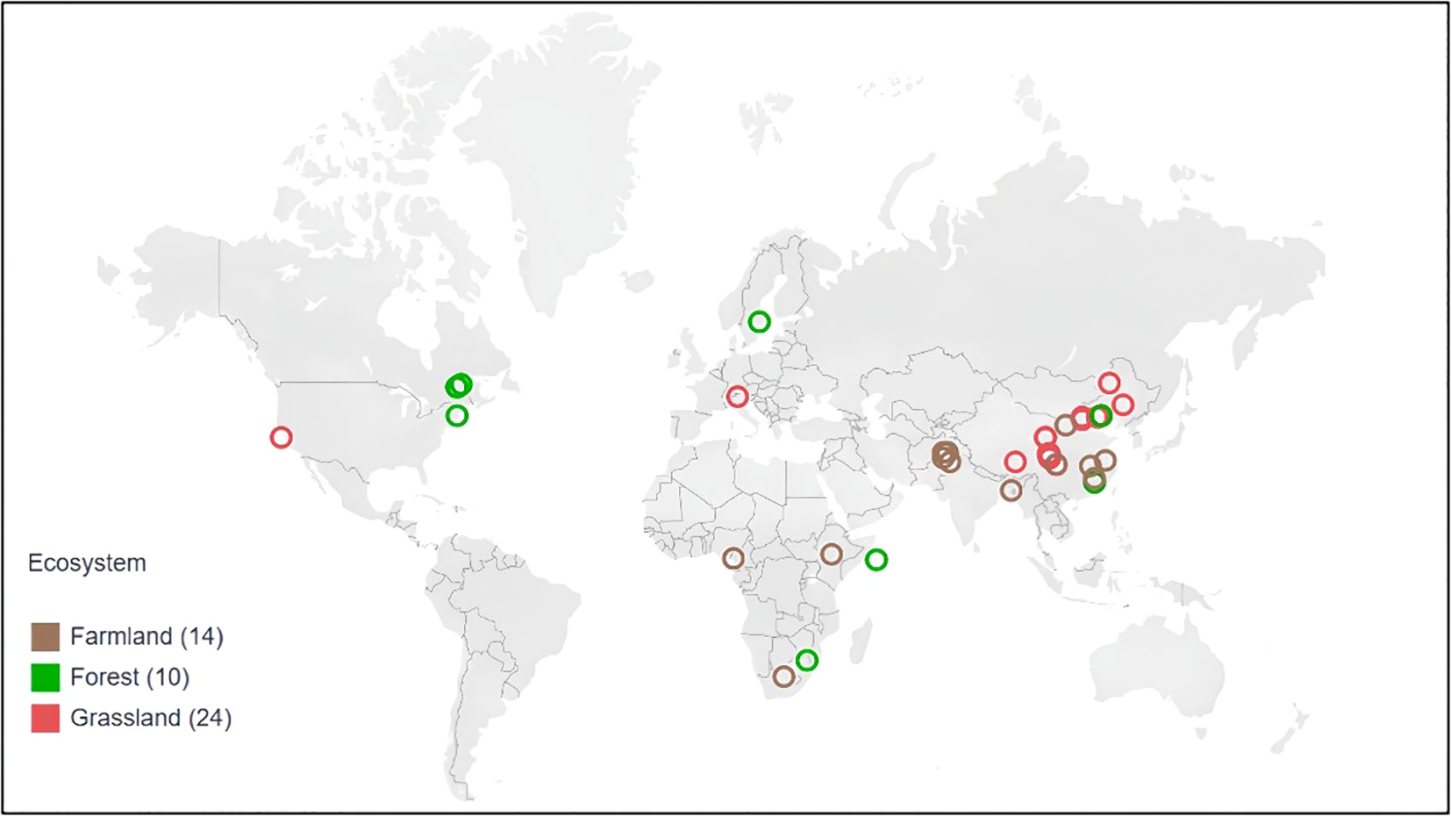
Global distribution of ecosystems in N addition experiments selected for meta-analysis.

For each study, we recorded the paper title, first author, year of publication, study location’s latitude and longitude, ecosystem type, phenological stage, nitrogen addition form, application rate, experiment duration, annual average precipitation and temperature, pH value, and the Latin name and family of each species studied. A total of 28 families and 125 species were included, with ecosystem types comprising forests, grasslands, farmlands (**Figure 1**).

### 2.4 Data processing and analysis

All meta-analyses were performed in R (version 4.4.1) using the “Metafor” package (version 4.6-0) (Viechtbauer, 2010; Team, 2014). A commonly used measure in meta-analysis is the response ratio (RR), which serves as an index to characterize the extent of an influence of a factor on a particular indicator. In this study, the RR reported in the literature was calculated using the Equation 2.


(2) $$RR=ln\frac{X_{e}}{X_{c}}$$

where Xe is the mean of the treatment and Xc is the mean of the control. In this study, the RR of the average phenological period for each species with and without nitrogen addition was calculated. Across all meta-analyses, the observed RR was weighted by the inverse of the sampling variance, which was calculated as Equation 3.


(3) $$V_{yi}=\frac{S_{e}^{2}}{N_{e}X_{e}^{2}}+\frac{S_{c}^{2}}{N_{C}X_{c}^{2}}$$

where Se is the SD of the treatment, Sc is the SD of the control, Ne is the sample size of the treatment, and Nc is the sample size of the control.

The meta-analysis was conducted in three steps. The overall effect of nitrogen application on plant phenology was calculated to determine whether it was significant. Considering that each study was conducted independently, considerable variation was observed. In this study, 582 pieces of data from 51 articles were divided into 10 typical phenological periods. As herbaceous plants and woody plants present very different growth processes, plant phenology was divided into 10 stages: budding, cessation, complete leaf fall, flowering, fruiting, leaf discoloration, maturity, and peak, senescence, and xylem growth. Additionally, we analyzed the effects of nitrogen addition on plant phenology in three different terrestrial ecosystems (forest, farmland, grassland ecosystems). To address data non-independence due to multiple effect sizes, a random-effects model meta-analysis was performed using the “Metafor” package in R. An effect size was considered significantly different from zero if the 95% confidence interval (CI) did not include zero and if *p*<0.05. The formal Cochran’s Q test (QE) was used to assess heterogeneity by evaluating whether the observed variability in effect sizes or outcomes exceeded what would be expected based solely on sampling variability. Continuous variables (mean annual temperature, nitrogen addition rate, and mean annual precipitation) were selected for a single mixed-effects model, and models with categorical factors were run without intercepts to obtain parameter estimates (mean effect size) for each level. The heterogeneity of each independent model was evaluated using the omnibus test. The R package “glmulti” was employed to determine the small-sample-corrected Akaike Information Criterion (AICc) for each model in the candidate set. In cases with multiple optimal models, the one with the lower AICc value was selected if the difference exceeded three.

Publication bias was evaluated using the funnel plot method. In the absence of bias, the effect size should not correlate with sample size, and a symmetrical funnel shape would suggest minimal bias. Statistical significance was set at *p*<0.05. We conducted Egger’s regression test to assess funnel plot asymmetry. Egger’s test for funnel plot asymmetry showed no statistical significance (*p*>0.05), indicating symmetrical distribution of studies and minimal risk of publication bias. Furthermore, Rosenberg’s fail-safe number was calculated, exceeding the critical value of 5k + 10 (where k represents the number of case studies), indicating that the findings are robust against publication bias. Additionally, Q-Q normal plots were used to assess model appropriateness by testing if residuals conform to a normal distribution, even though typical data may not follow this distribution. Herein, the points were within the CI range, indicating good model fit.

## 3 Results

In this study, we analyzed the response of plant phenology to nitrogen addition across different ecosystems from a comprehensive and quantitative perspective. Our results indicate that the effect of nitrogen addition on plant phenological stages varies depending on the ecosystem type. This study also found that environmental factors regulated the response of plant phenological stages to nitrogen addition in different ecosystems.

### 3.1 Varying extent of nitrogen addition on phenological phases

**Figure 2:**
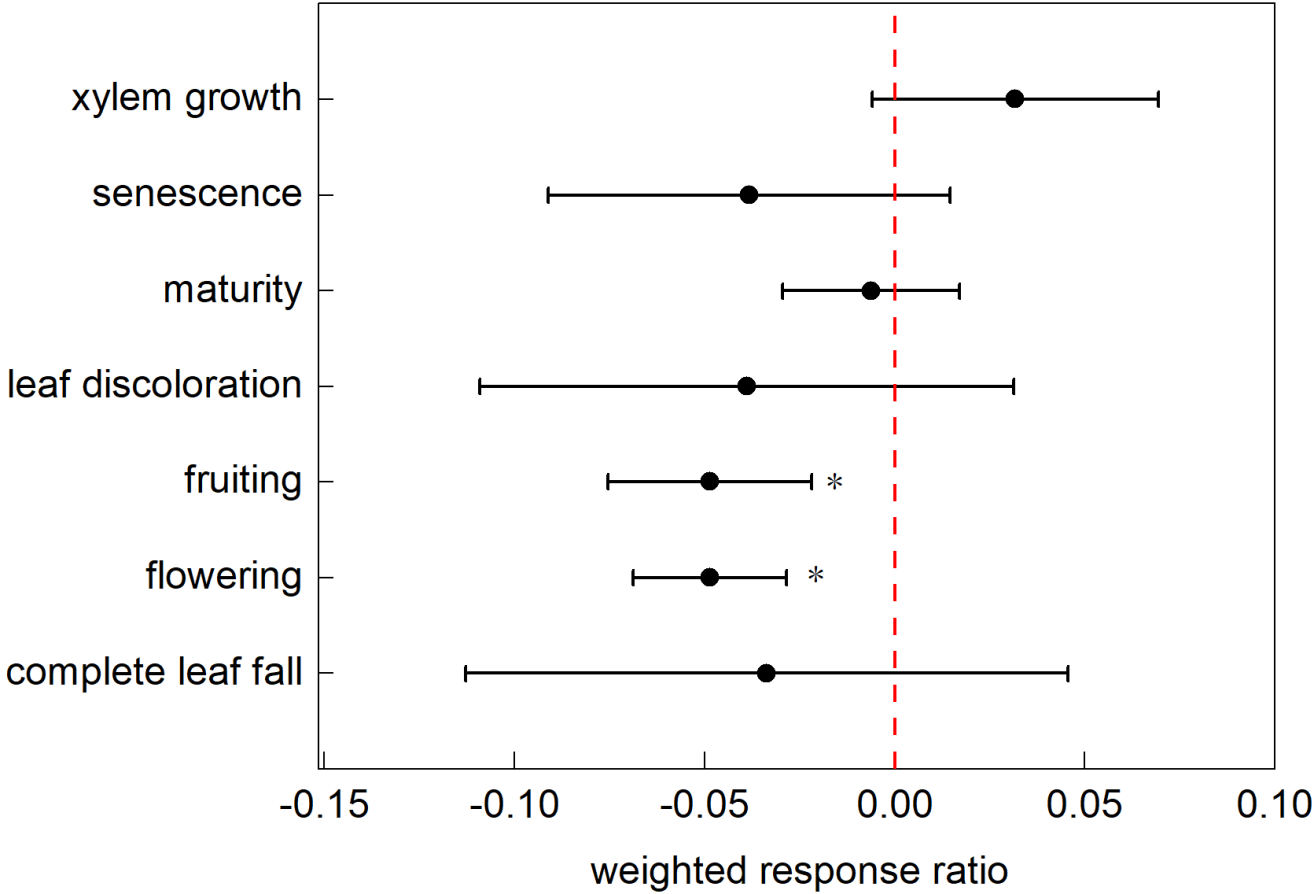
Response ratios of different phenological stages to nitrogen addition. Note: Points with error bars represent the overall mean response ratio, with a 95% confidence interval (CI). *indicates statistical significance (p<0.05).

This study analyzed 582 data points from 51 studies categorized into 10 typical phenological phases. The analysis yielded a 95% CI of (0.0070, 0.0171), SE of 0.0026, and *p*-value<0.0001, indicating significant correlation. The confidence interval (CI) excluding zero with a narrow range indicates a statistically significant effect size; the small standard error (SE) demonstrates minimal deviation between the sample mean and population mean, reflecting high reliability of the estimate; and the *p*-value<0.05 provides robust evidence of exceptionally strong statistical significance in the study findings. Therefore, the impact of nitrogen addition on the phenology of terrestrial ecosystems was significant within the study range. However, the effects of nitrogen addition varied across phenological stages. The results showed that only the complete leaf fall stage did not respond significantly to nitrogen addition, whereas all other phenological stages showed significant responses to nitrogen addition (**Figure 2**).

**Figure 3:**
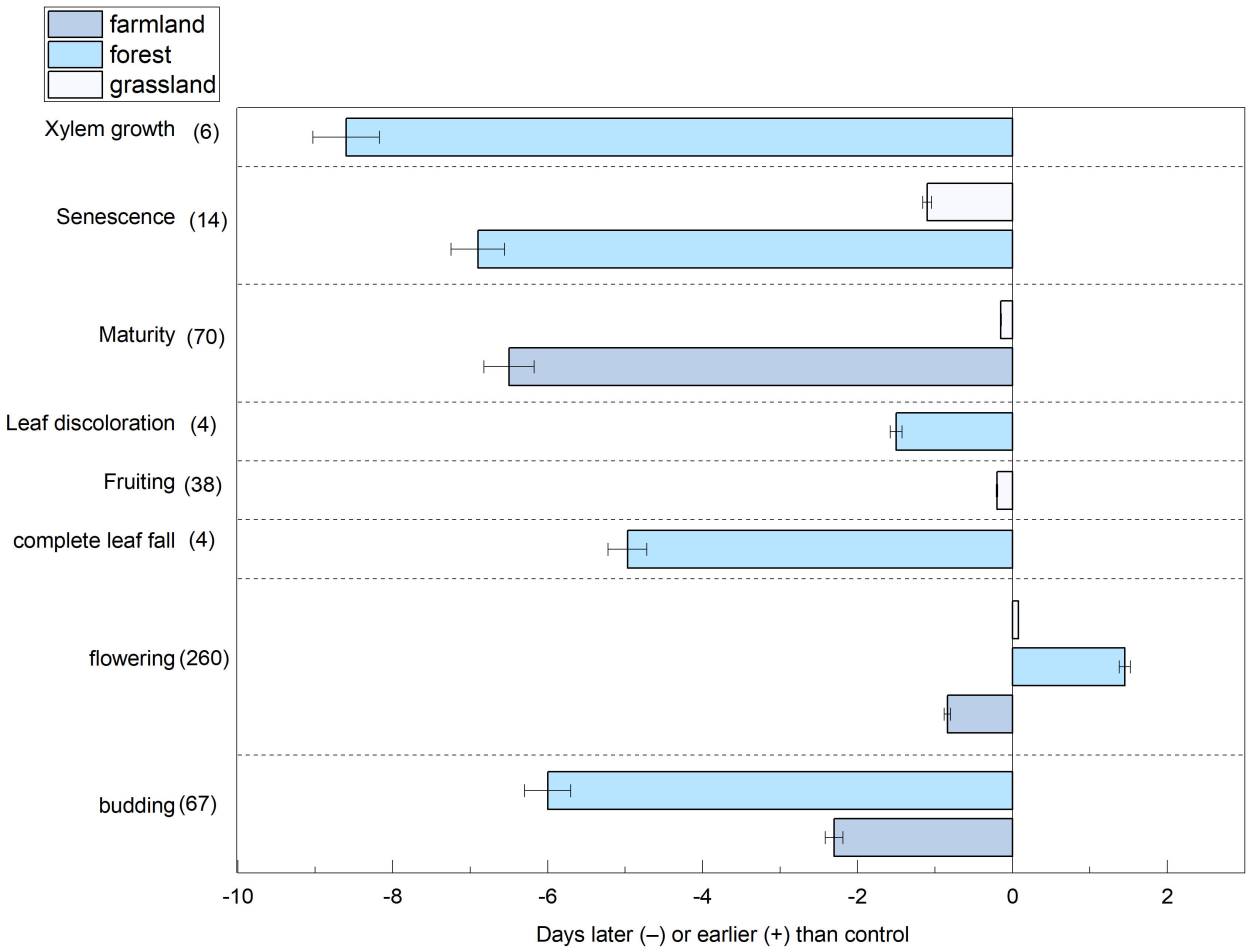
The effects of nitrogen addition on budding, flowering, complete leaf fall, fruiting, leaf discoloration, maturity, senescence and xylem growth phenology in three ecosystems, the sample size is indicated in parentheses.

Our findings indicated that nitrogen addition advanced the flowering phase, with an average advancement of 0.18 d. In contrast, the phenological phases of budding, complete leaf fall, fruiting, leaf discoloration, maturity, senescence, and xylem growth were delayed under nitrogen addition, with average delays of 4.15, 4.95, 0.21, 1.5, 3.3, 4, and 8.56 d, respectively (**Figure 3**).

### 3.2 Effects of nitrogen addition on phenology vary across different ecosystems

**Figure 4:**
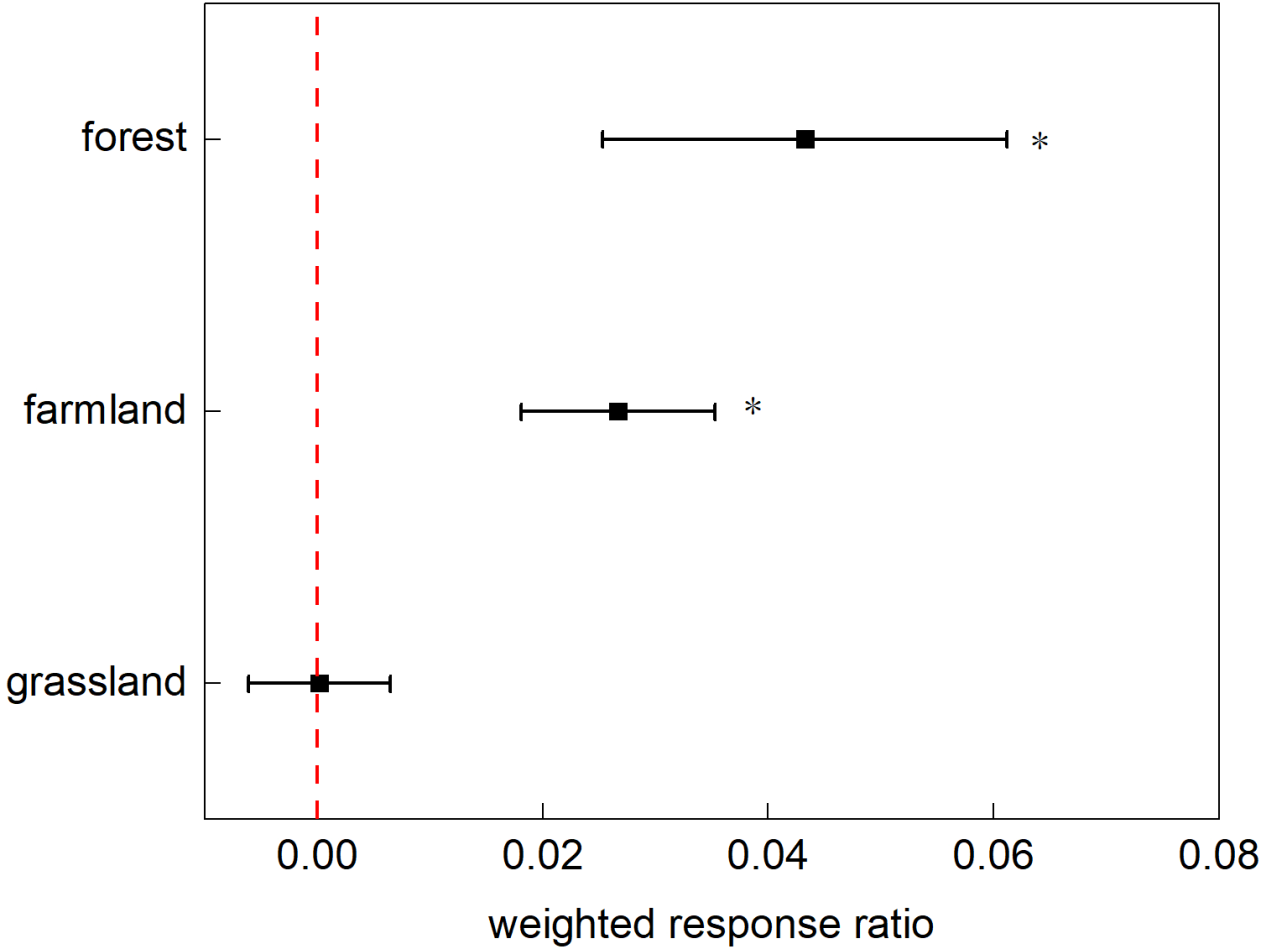
The response ratio of different ecosystems’ phenological stages to nitrogen addition. Points with error bars represent the overall average response ratio, with a 95% confidence interval (CI). An asterisk (*) denotes statistical significance (p<0.05).

This study calculated the effects specific to each ecosystem using a random-effects model (**Figure 4**). Nitrogen addition significantly affected plant phenology in the farmland and tundra ecosystems. In the farmland ecosystem, all plant phenological phases were affected, with the onset of phenological events delayed by an average of 3.21 d (*p*<0.0001; 95% CI: 2.857, 4.0353). In the forest ecosystem, plants exhibited varied responses to nitrogen addition during different phenological phases (*p*<0.0001), with budding advancing, whereas the other stages were delayed. Nitrogen application had a weaker effect on grassland ecosystems, with phenological events delayed by an average of 0.34 d.

### 3.3 Analysis of other factors influencing phenological responses

**Figure 5:**
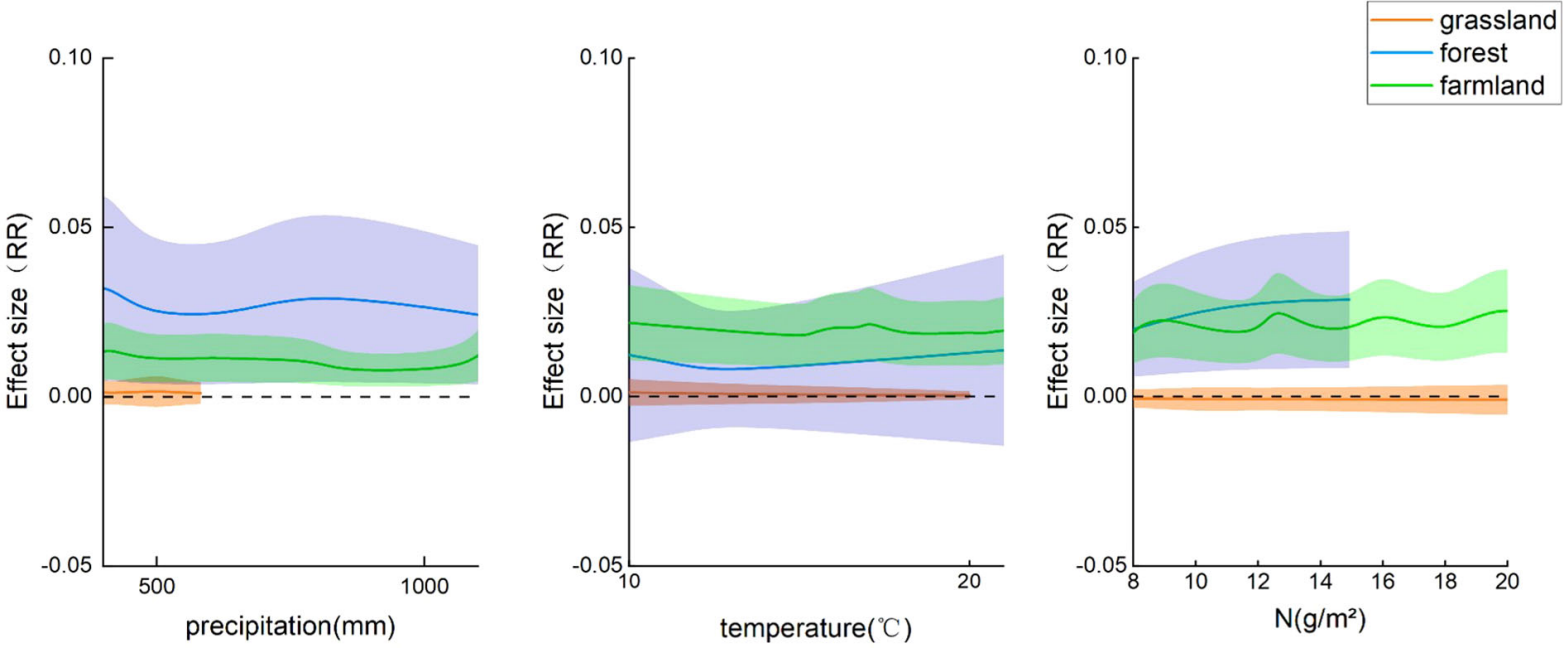
Changes in effect values of different ecosystems with varying influencing factors. RR, effect value, shaded areas represent the confidence intervals of effect values for different ecosystems.

The phenological phases of plants are affected by nitrogen addition and various environmental factors. To better explain the changes in plant phenology, this study introduced three influencing factors (annual average temperature, annual average precipitation, and nitrogen addition rate) to elucidate the impact of nitrogen addition on phenological phases. Using a mixed-effects model to calculate the influence of these three factors on phenological phases, all three factors were significantly correlated. Nitrogen addition rate (*p*<0.001; 95% CI: -0.0005, 0.0011), annual average temperature (*p*<0.001; 95% CI: 0.0004, 0.0014), and annual average precipitation (*p*<0.001; 95% CI: 0.0000, 0.0000). Precipitation had the most substantial impact on phenological phases in forest and farmland ecosystems, with a relatively smaller impact on grassland ecosystems. Temperature and nitrogen addition rate had a more intense influence on the effect value for forest ecosystems than for the other two ecosystems (**Figure 5**).

**Figure 6:**
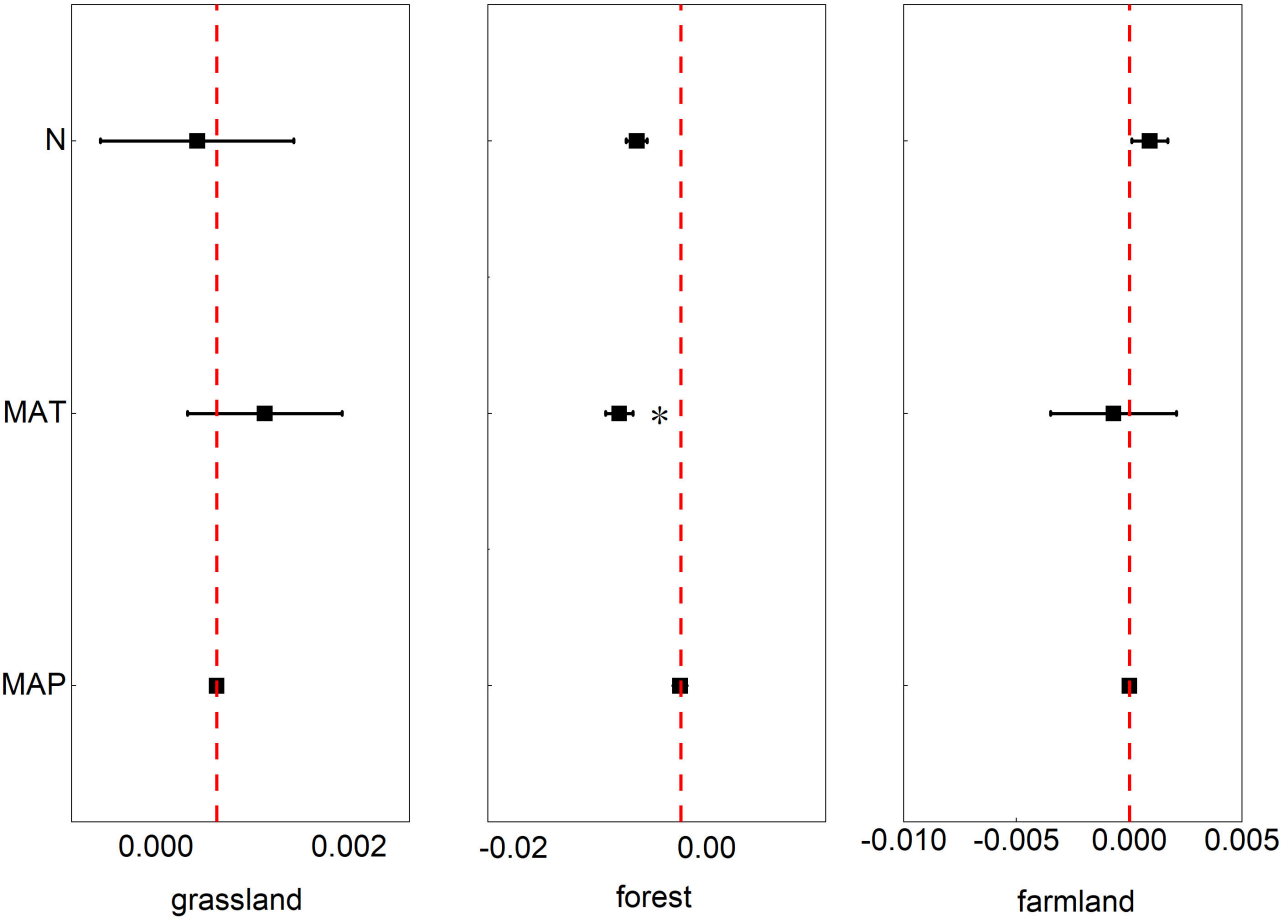
The effects of nitrogen addition rate (N), mean annual temperature (MAT), and mean annual precipitation (MAP) on the phenology of grassland ecosystems, farmland ecosystems, and forest ecosystems. Note: Points with error bars represent the overall mean response ratio, with a 95% confidence interval (CI). * indicates statistical significance (p<0.05).

According to our analyses, temperature had a significant effect on the phenological phases in the forest ecosystem (*p*<0.05, 95% CI: -0.0115, -0.0014). In the farmland ecosystem, these three influencing factors had a slight impact on the phenological phases. However, the phenological phases of the grassland ecosystem were not significantly affected by these three factors (**Figure 6**).

## 4 Discussion

### 4.1 Impact of nitrogen addition on the phenological phases across different ecosystems

Most phenological phases were delayed under the influence of nitrogen deposition. However, the flowering phase in grassland and forest ecosystems advanced with increasing nitrogen levels.

Nitrogen is a primary limiting factor for plant growth in terrestrial ecosystems; therefore, nitrogen addition affects plant functional traits and reproductive strategies, significantly influencing plant phenology. In forest ecosystems, nitrogen addition to the canopy enhances soil nutrient availability, leading to increased nitrogen content in plant buds, enhancing the absorption of nutrients by roots and thereby increasing leaf nitrogen concentration, which aids in the synthesis of chlorophyll and proteins (Tian et al., 2024). The addition of nitrogen significantly enhances photosynthesis by increasing chlorophyll content and leaf area, allowing plants to accumulate more carbohydrates, promote growth and development, and potentially lead to earlier flowering. Enhancing photosynthesis affects plant hormones (e.g., gibberellins and auxins) synthesis and signaling, further promoting flowering. As photosynthesis increases, plants accumulate more resources, delaying leaf senescence and extending the growing season (Zhou and Yang, 2023). Under conditions of resource abundance, plants delay the allocation shift from growth to reproduction, thereby postponing phenological development (Stinziano et al., 2015; Zhou et al., 2023). In nitrogen-limited ecosystems, resource availability often leads plants to allocate more resources to growth rather than reproduction, potentially delaying the transition from growth to the reproductive stage (Arroyo-Rodriguez et al., 2020). This can lead to significant delays in budburst and subsequent phenological stages. Further studies have shown that nitrogen input into forest ecosystems delays plant budburst, and a clear delay in the initiation of the growing season was observed in trees that had been treated with nitrogen for 3 years (Kula et al., 2012). A previous study on the grassland plant alfalfa found that nitrogen application delayed the initial flowering stage, advanced the final flowering stage, reduced the overall flowering period (Ma et al., 2023).

Studies have also shown that in addition to increasing nitrogen availability, nitrogen addition affects the plant growth hormones that regulate plant phenology, thereby delaying leaf growth. Excessive nitrogen increase can limit the production of cytokinins while increasing the production of abscisic acid. When the negative impact of growth hormones on the growth of plant leaves exceeds the positive effects of nutrient availability, then delayed leaf growth occurs This study demonstrates that nitrogen addition in forest ecosystems induces earlier flowering in plants through enhanced nitrogen availability, which accelerates reproductive development by optimizing nutrient allocation, consequently advancing the phenological phase (Zhou et al., 2023). Similarly, nitrogen addition on North American prairies advances flowering in some herbaceous plants. Earlier flowering in these treatments is a strategy to avoid competition for resources, allowing plants to ensure reproductive growth and future survival in the community (Silva et al., 2021). This reproductive strategy represents an adaptation to environments that receive supplemental nitrogen (Wang and Tang, 2019).

### 4.2 Influencing factors regulate the response of plant phenology to nitrogen deposition in different ecosystems

The ability of plants to respond to environmental factors that influence phenological periods is a key strategy for coping with the spatiotemporal heterogeneity of their environment (Fay et al., 2018). In forest ecosystems, research has indicated that individual nitrogen enrichment is neither beneficial for vegetation growth nor a source of stress. Instead, environmental factors interact with nitrogen addition to affect plant growth (Wheeler et al., 2017a). Further studies have found that warming soil and air significantly engender an earlier phenological phase (Dao et al., 2015; Wheeler et al., 2017b), which is reflected in the nearly 3-week delay in the start time of budding in late spring compared to that of the previous years. Additionally, higher temperatures after a delay in the germination period are conducive to leaf unfolding (Bigler and Vitasse, 2019), suggesting that temperature is a potential mechanism driving the advancement of phenological phases in forest ecosystems. Research on forest plants has also demonstrated that nitrogen addition can delay germination. For instance, nitrogen-treated birch trees showed delayed germination (Kula et al., 2012), with soil temperature and nitrogen availability playing crucial roles in this process. Previous studies on farmland ecosystem phenology have identified that, among environmental factors, temperature is the primary driver of phenological changes in rice (Chen et al., 2021). Additionally, temperature has a significant impact on wheat phenology as well (Yang et al., 2023a). Owing to the length of the growing season in crops is related to cumulative and minimum temperatures, phenological phenomena occurs only after certain thermal requirements have been met (Cesaraccio et al., 2001; Wang et al., 2008).

Temperature promotes plant phenological changes through a complex physiological and ecological process. Plants require the accumulation of a certain amount of effective accumulated temperature (the thermal sum required for developmental stages) to complete specific phenological stages, such as leaf unfolding or flowering. Rising temperatures accelerate enzymatic activity (e.g., starch hydrolases and photosynthetic enzymes), thereby promoting cell division and differentiation. This physiological response may ultimately shorten the phenological cycle (Wheeler et al., 2017a). Studies in China’s warm temperate zone show that the onset of leaf unfolding and flowering in woody plants advances by 0.23–4.96 days per decade, significantly correlated with spring accumulated temperature. In the Northern Hemisphere, daytime maximum temperatures (compared to daily mean temperatures) play a more critical role in triggering spring leaf-out, highlighting the importance of diurnal temperature variation. Temperature interacts with other environmental factors to shape plant phenology. In arid regions, the combined effect of rising temperatures and reduced precipitation induces soil moisture stress, which delays phenological phases. For example, alpine meadows on the Tibetan Plateau exhibit delayed green-up periods primarily due to this mechanism (Yang et al., 2023b). Conversely, when moderate warming coincides with adequate precipitation, their synergistic effect markedly accelerates phenological progression.

Unlike temperature, the delaying effect of nitrogen addition on plant bud germination has been inconsistent, although its long-term effect is more pronounced. The response of woody forest species to nitrogen addition varies. Researchers have shown that precipitation can alleviate water stress, causing a delay in the autumn phenology of forest vegetation (Liu et al., 2016). The distribution of precipitation strongly affects changes in phenological timing, and the amount of precipitation and nitrogen addition have a significant positive impact on the phenology of plant growth periods. Compared to moderate levels, high levels of water and nitrogen addition have an insignificant impact on phenological phases (Moyo et al., 2015). Thus, each influencing factor acts in conjunction with plant growth.

Researchers conducted multiple nitrogen addition experiments in arid, semi-arid, and semi-humid ecosystems and discovered that the relative response of crops to nitrogen did not increase with increased precipitation. This could be because precipitation enhances soil nitrogen mineralization (Peng et al., 2024) or increases rhizosphere nitrogen leaching, resulting in similar soil inorganic nitrogen contents in both the nitrogen addition and control plots. Other researchers suggested that multilevel nitrogen application does not significantly affect plant phenology (Xi et al., 2015). Similar studies have reported a minimal effect of varying nitrogen supply levels on plant developmental timing (Zhang et al., 2022). Plant species composition underlies variation in responses to nitrogen addition gradients, with distinct functional groups exhibiting differential nitrogen-use efficiencies that drive ecosystem-level allocation patterns (Su et al., 2021); however, the extent to which phenology responds to multilevel nitrogen addition remains unclear. Certain grassland ecosystems, such as alpine meadow systems on the Qinghai–Tibet Plateau, are also limited by nitrogen availability, partly because of slow nitrogen mineralization (Xi et al., 2015). Qi found that the peak growth rate of Leymus chinensis was significantly delayed by increased precipitation, and other researchers observed that dominant perennial plants in communities are more sensitive to photoperiod than forb species, with plants commencing phenological activity only after reaching the critical photoperiod (Xia and Wan, 2013).

This study found that the impact of nitrogen deposition on plant phenology is more significant in farmland ecosystems than in grassland ecosystems. The experimental results of this study confirm that community-level biodiversity enhancement mitigates nitrogen deposition impacts on plant phenology, with species-rich assemblages buffering phenological shifts through complementary nutrient-use strategies (Xia and Wan, 2013; Li et al., 2024). A phenological observational study performed over four growing seasons in grassland ecosystems indicated no interactive effect of temperature and nitrogen addition on phenological events in any species, possibly because of species-specific responses in plant phenology. The effect of temperature increase was induced by certain species. Furthermore, the response to temperature changes varies among different flowering functional groups, with early-flowering plants being more sensitive to warming than late-flowering plants, indicating that the delaying effect of nitrogen is independent of specific growth forms. Phenological observations in alpine meadows on the Qinghai–Tibet Plateau have demonstrated that vegetation growth is strongly limited by nitrogen availability, partly because of the slow rate of nitrogen mineralization. Consequently, nitrogen application has minimal effects on grassland ecosystems (Peng et al., 2024). Therefore, the determinants of plant phenological activity vary across different periods, and plant growth patterns are influenced by the collective effects of factors such as temperature, precipitation, and light (Qi, 2023).

In environments with nitrogen addition, temperature has a more pronounced regulatory effect on the phenology of forest ecosystems but a minimal impact on grassland and agricultural ecosystems. Nitrogen addition drives shifts in nitrogen-use efficiency and carbon allocation patterns, while mediating root-shoot interactions, which collectively enhance the temperature sensitivity of phenological processes in forest ecosystems through modified plant-soil feedbacks (Bicharanloo et al., 2022). In contrast, the phenology of grassland and agricultural ecosystems is more dominated by short-term environmental factors such as moisture and light, and their open structure and shallow root systems weaken the core regulatory role of temperature. This difference reflects fundamental distinctions in functional types, resource allocation, and climate adaptation strategies among different ecosystems.

## 5 Conclusion

We systematically analyzed the response of plant phenology to nitrogen addition across different terrestrial ecosystems on a global scale. Our results indicate that nitrogen addition affects different phenological stages in various ways, with responses to phenology varying across ecosystems. For instance, the flowering phenology in forest ecosystems was advanced 1.45d under the influence of nitrogen deposition, whereas the Budding, Flowering, Fruiting, Maturity phenology in grassland ecosystem and farmland ecosystem were delayed by nitrogen addition. Moreover, temperature, precipitation, and nitrogen addition did not significantly regulate the phenological periods of plants in grassland and farmland ecosystems, while temperature had a relatively significant effect on the phenological periods in forest ecosystems. In summary, nitrogen addition has substantial impacts on plant phenology in terrestrial ecosystems, with effects varying across ecosystem types and environmental conditions. This study clarifies the overall impact direction of nitrogen addition on plant phenology, establishes a unified theoretical framework for understanding the ecological effects of nitrogen deposition, and provides a theoretical foundation for predicting ecosystem succession under global change. Future research should carefully consider the combined effects of these factors on plant phenology by establishing an integrated phenological monitoring technology system and a cross-ecosystem observation network.

## Data Availability

The original contributions presented in the study are included in the article/**Supplementary Material**. Further inquiries can be directed to the corresponding author.
