# Supplementary material for: Nitrogen addition substantially affects plant phenology in terrestrial ecosystems: a meta-analysis
Source: Front Plant Sci. 2025 Aug 19;16:1632357. doi: 10.3389/fpls.2025.1632357 (PMC12401924; doi:10.3389/fpls.2025.1632357)
Supplement: Supplementary file 1 [file DataSheet1.docx]

Supplementary Material

# 1 Supplementary Data

# Data 1. The list of 51 papers from which the data were extracted for this meta-analysis

Ali, K., Arif, M., Shah, S., Hussain, Z., Ali, A., Munir, S., et al. (2015). EFFECT OF ORGANIC AND INORGANIC NUTRIENTS SOURCES ON PHENOLOGY AND GROWTH OF WHEAT. Pakistan Journal of Botany 47(6), 2215-2222.

Amanullah, Khattak, R.A., and Khalil, S.K. (2009). Plant Density and Nitrogen Effects on Maize Phenology and Grain Yield. Journal of Plant Nutrition 32(2), 246-260. doi: 10.1080/01904160802592714.

Azeem, K., Khalil, S., Khan, F., Shahenshah, S., Qahar, A., Sharif, M.N., et al. (2014). Phenology, Yield and Yield Components of Maize as Affected by Humic Acid and Nitrogen. 6, 286.

Basir, A., Jan, M.T., Arif, M., and Khan, M.J. (2016). Response of Tillage, Nitrogen and Stubble Management on Phenology and Crop Establishment of Wheat. International Journal of Agriculture and Biology 18(1), 1-8.

Cleland, E.E., Chiariello, N.R., Loarie, S.R., Mooney, H.A., and Field, C.B. (2006). Diverse responses of phenology to global changes in a grassland ecosystem. Proc Natl Acad Sci U S A 103(37), 13740-13744. doi: 10.1073/pnas.0600815103.

Cronje, R., and Ratlapane, I. (2018). Effect of various N application times on tree phenology and physiology of ‘Mauritius’ litchi in South Africa – preliminary results. Acta Horticulturae 1211, 7-14. doi: 10.17660/ActaHortic.2018.1211.2.

Dao, M.C.E., Rossi, S., Walsh, D., Morin, H., and Houle, D. (2015). A 6-Year-Long Manipulation with Soil Warming and Canopy Nitrogen Additions does not Affect Xylem Phenology and Cell Production of Mature Black Spruce. Frontiers in Plant Science 6. doi: 10.3389/fpls.2015.00877.

Fang, Y., Li, H., and Liao, S. (2019). Effects of nitrogen and phosphorus application rate on flowering characteristics of oilseed rape. Chinese Journal of Oil Crop Sciences 41(2), 199. doi: 10.7505/j.issn.1007-9084.2019.02.006.

Fatima, S., Khan, M.S., Nadeem, M., Khan, I., Waseem, K., Nisar, M., et al. (2019). Interactive Effects of Genotype and Nitrogen on the Phenology and Yield Determination of Okra <i>Abelmoschus esculentus</i> (L.). International Journal of Plant Production 13(1), 73-90. doi: 10.1007/s42106-019-00035-x.

Fu, Y.S.H., Piao, S.L., Delpierre, N., Hao, F.H., Hänninen, H., Geng, X.J., et al. (2019). Nutrient availability alters the correlation between spring leaf-out and autumn leaf senescence dates. Tree Physiology 39(8), 1277-1284. doi: 10.1093/treephys/tpz041.

Gungula, D., Togun, A., and Kling, J. (2007). The effects of nitrogen rates on phenology and yield components of early maturing maize cultivars. Global Journal of Pure and Applied Sciences 13. doi: 10.4314/gjpas.v13i3.16711.

Haque, S., and Khaliq, Q. (2006). Effect of Nitrogen on Phenology, Light Interception and Growth in Aromatic Rice.

Huang, G., Li, C.H., and Li, Y. (2018). Phenological responses to nitrogen and water addition are linked to plant growth patterns in a desert herbaceous community. Ecology and Evolution 8(10), 5139-5152. doi: 10.1002/ece3.4001.

Ibrahim, M., and Khan, A. (2017). Phenology and Maize Crop Stand in Response to Mulching and Nitrogen Management. Sarhad Journal of Agriculture 33. doi: 10.17582/journal.sja/2017/33.3.426.434.

Imranuddin, Arif, M., Khan, A., Sadiq, M., Ahmed, I., Ahmed, N., et al. (2016). Seed Priming, Nitrogen Levels and Moisture Regimes Affects Phenology of WheatSeed Priming, Nitrogen Levels and Moisture Regimes Affects Phenology of Wheat. European Academic Research IV, 5563-5582.

Khan, M., Sajid, M., Hussain, Z., Rab, A., Marwat, K., Fazal, I.W., et al. (2013). How nitrogen and phosphorus influence the phenology of OKRA. Pakistan Journal of Botany 45, 479-482.

Khan, Z.H., Khalil, S.K., Iqbal, A., Ullah, I., Ali, M., Shah, T., et al. (2017). NITROGEN DOSES AND PLANT DENSITY AFFECT PHENOLOGY AND YIELD OF SWEET CORN. Fresenius Environmental Bulletin 26(6), 3809-3815.

Kou, L., Li, S.G., Wang, H.M., Fu, X.L., and Dai, X.Q. (2019). Unaltered phenology but increased production of ectomycorrhizal roots of <i>Pinus elliottii</i> under 4 years of nitrogen addition. New Phytologist 221(4), 2228-2238. doi: 10.1111/nph.15542.

Liu, L., Monaco, T.A., Sun, F.D., Liu, W., Gan, Y.M., and Sun, G. (2017a). Altered precipitation patterns and simulated nitrogen deposition effects on phenology of common plant species in a Tibetan Plateau alpine meadow

Agricultural and Forest Meteorology 236, 36-47. doi: 10.1016/j.agrformet.2017.01.010.

Liu, X., Zhang, Z., and Du, G. (2021). Response of flowering phenology of main components to nitrogen addition in alpine meadow. Pratacultural Science 38(07), 1240-1249.

Liu, Y.Z., Miao, R.H., Chen, A.Q., Miao, Y., Liu, Y.J., and Wu, X.W. (2017b). Effects of nitrogen addition and mowing on reproductive phenology of three early-flowering forb species in a Tibetan alpine meadow. Ecological Engineering 99, 119-125. doi: 10.1016/j.ecoleng.2016.11.033.

Liu, Z.C., Fu, Y.H., Shi, X.R., Lock, T.R., Kallenbach, R.L., and Yuan, Z.Y. (2022). Soil moisture determines the effects of climate warming on spring phenology in grasslands. Agricultural and Forest Meteorology 323. doi: 10.1016/j.agrformet.2022.109039.

Ma, T., Zeng, W.Z., Li, Q., Wu, J.W., and Huang, J.S. (2016). Effects of water, salt and nitrogen stress on sunflower Helianthus annuus at different growth stages. Journal of Soil Science and Plant Nutrition 16(4), 1024-1037.

Ma, Y., Zhao, X., Li, X., Hu, Y., and Wang, C. (2023). Intraspecific Variation in Functional Traits of Medicago sativa Determine the Effect of Plant Diversity and Nitrogen Addition on Flowering Phenology in a One-Year Common Garden Experiment. Plants-Basel 12(10). doi: 10.3390/plants12101994.

Mahama, G.Y., Prasad, P.V.V., Roozeboom, K.L., Nippert, J.B., and Rice, C.W. (2016). Cover Crops, Fertilizer Nitrogen Rates, and Economic Return of Grain Sorghum. Agronomy Journal 108(1), 1-16. doi: 10.2134/agronj15.0135.

Marty, C., Piquette, J., Dussault-Chouinard, E., Morin, H., Thiffault, N., Houle, D., et al. (2020). Canopy Nitrogen Addition and Soil Warming Affect Conifer Seedlings' Phenology but Have Limited Impact on Growth and Soil N Mineralization in Boreal Forests of Eastern Canada. Frontiers in Forests and Global Change 3. doi: 10.3389/ffgc.2020.581363.

Moyo, H., Scholes, M.C., and Twine, W. (2015). Effects of water and nutrient additions on the timing and duration of phenological stages of resprouting Terminalia sericea. South African Journal of Botany 96, 85-90. doi: 10.1016/j.sajb.2014.10.009.

Peng, Y., Yang, J.X., Zhou, X.H., Peng, P.H., Li, J.J., and He, W.M. (2018). Warming delays the phenological sequences of an autumn-flowering invader. Ecology and Evolution 8(12), 6299-6307. doi: 10.1002/ece3.4177.

Peng, Y., Yang, J.X., Zhou, X.H., Peng, P.H., Li, J.J., Zhang, S.M., et al. (2019). An invasive population of <i>Solidago canadensis</i> is less sensitive to warming and nitrogen-addition than its native population in an invaded range. Biological Invasions 21(1), 151-162. doi: 10.1007/s10530-018-1812-2.

Petraglia, A., Tomaselli, M., Mondoni, A., Brancaleoni, L., and Carbognani, M. (2014). Effects of nitrogen and phosphorus supply on growth and flowering phenology of the snowbed forb <i>Gnaphalium supinum</i> L. Flora 209(5-6), 271-278. doi: 10.1016/j.flora.2014.03.005.

Raj, A., Chakrabarti, B., Pathak, H., Singh, S.D., Mina, U., and Mittal, R. (2016). Growth, yield components and grain yield response of rice to temperature and nitrogen levels. Journal of Agrometeorology 18(1), 1-6.

Sayyad-Amin, P. (Year). "The study of sesame phenology and growth period under different nitrogen rates").

Sharifi, R.S., and Namvar, A.J.B. (2016). Effects of time and rate of nitrogen application on phenology and some agronomical traits of maize (Zea mays L.). 62.

Sharma, N., Sinha, V.B., Kumar, N.A.P., Subrahmanyam, D., Neeraja, C.N., Kuchi, S., et al. (2021). Nitrogen Use Efficiency Phenotype and Associated Genes: Roles of Germination, Flowering, Root/Shoot Length and Biomass. Frontiers in Plant Science 11. doi: 10.3389/fpls.2020.587464.

Smith, J.G., Sconiers, W., Spasojevic, M.J., Ashton, I.W., and Suding, K.N. (2012). Phenological Changes in Alpine Plants in Response to Increased Snowpack, Temperature, and Nitrogen. Arctic Antarctic and Alpine Research 44(1), 135-142. doi: 10.1657/1938-4246-44.1.135.

Tadesse, A., and Kim, H.K. (2015). Yield Related Traits and Yield of Quality Protein Maize (Zea mays L.) affected by Nitrogen Levels to achieve maximum yield in the Central Rift Valley of Ethiopia. 5, 15.

Tian, L. (2023). Effects of climate warming and nitrogen deposition on plant phenology in temperate steppe. Master.

Tian, L., Zhu, Y., Li, X., Han, G.-D., and Ren, H.-Y. (2022). Responses of plant phenology to warming and nitrogen addition under different precipitation conditions in a desert steppe of Nei Mongol, China. Chinese Journal of Plant Ecology 46(3), 290-299. doi: 10.17521/cjpe.2021.0277.

Tigre, W., Worku, W., and Haile, W. (2014). Effects of Nitrogen and Phosphorus Fertilizer Levels on Growth and Development of Barley (Hordeum vulgare L.) at Bore District, Southern Oromia, Ethiopia. American Journal of Life Sciences 2, 260-266. doi: 10.11648/j.ajls.20140205.12.

Wang, P.L., Fu, C., Wang, L.Y., and Yan, T. (2022). Delayed autumnal leaf senescence following nutrient fertilization results in altered nitrogen resorption. Tree Physiology 42(8), 1549-1559. doi: 10.1093/treephys/tpac028.

Wang, Y. (2019). Shifts in plant reproductive phenology under global change factors in a Tibetan alpine meadow. Master

Wani, S., Qayoom, S., Bhat, M., Sheikh, A., Bhat, T., and Hussain, S. (2017). Effect of Varying Sowing Dates and Nitrogen Levels on Growth and Physiology of Scented Rice. ORYZA 54, 97-106.

Weih, M. (2009). Genetic and environmental variation in spring and autumn phenology of biomass willows effects on shoot growth and nitrogen economy. Tree Physiology 29(12), 1479-1490. doi: 10.1093/treephys/tpp081.

Wheeler, J., Frey, S., and Stinson, K. (2017). Tree seedling responses to multiple environmental stresses: Interactive effects of soil warming, nitrogen fertilization, and plant invasion. Forest Ecology and Management 403, 44-51. doi: 10.1016/j.foreco.2017.08.010.

Xi, Y., Zhang, T., Zhang, Y.J., Zhu, J.T., Zhang, G.L., and Jiang, Y.B. (2015). Nitrogen addition alters the phenology of a dominant alpine plant in northern Tibet. Arctic Antarctic and Alpine Research 47(3), 511-518. doi: 10.1657/aaar0014-054.

Xia, J.Y., and Wan, S.Q. (2013). Independent effects of warming and nitrogen addition on plant phenology in the Inner Mongolian steppe. Annals of Botany 111(6), 1207-1217. doi: 10.1093/aob/mct079.

Xu, X.T., Hu, G.Z., Liu, X., Lu, S.W., Li, S.N., and Zhao, N. (2021). Impacts of nitrogen enrichment on vegetation growth dynamics are regulated by grassland degradation status. Land Degradation & Development 32(14), 4056-4066. doi: 10.1002/ldr.3899.

Yang, C.R., Wei, S.B., Han, N., Liu, M.T., Mao, R.L., and Chen, C.Q. (2023a). Linear dependency of winter wheat yield and nitrogen use efficiency on the pre-anthesis temperature in the lower reach of the Yangtze River. European Journal of Agronomy 145. doi: 10.1016/j.eja.2023.126773.

Yang, X., Guo, R., Knops, J.M.H., Mei, L.L., Kang, F.R., Zhang, T., et al. (2020). Shifts in plant phenology induced by environmental changes are small relative to annual phenological variation. Agricultural and Forest Meteorology 294. doi: 10.1016/j.agrformet.2020.108144.

Yang, X.R., Chen, Y.Y., Zhang, T.W., Zhang, P.H., Guo, Z.P., Hu, G.R., et al. (2023b). Different responses of functional groups to N addition increased synchrony and shortened community reproductive duration in an alpine meadow. Journal of Ecology 111(10), 2231-2244. doi: 10.1111/1365-2745.14173.

Yang, Y., Zhang, H., Zhou, H., Ye, X., Yao, B., Zhang, C., et al. (2015). Short-term responses of flowering phenology and community structure to nitrogen, phosphorus and potassium in an alpine meadow on the Qinghai-Tibetan Plateau. Acta Prataculturae Sinica 24(8), 35-43.

Yin, T.F., Zheng, L.L., Cao, G.M., Song, M.H., and Yu, F.H. (2017). Species-specific phenological responses to long-term nitrogen fertilization in an alpine meadow. Journal of Plant Ecology 10(2), 301-309. doi: 10.1093/jpe/rtw026.

Zhang, Z. (2013). Effects of nitrogen addition on flowering phenology and community structure of alpine meadow community in eastern margin of Qinghai-Tibet Plateau

Doctor.

Zhang, Z.L., Niu, K.C., Liu, X.D., Jia, P., and Du, G.Z. (2014). Linking flowering and reproductive allocation in response to nitrogen addition in an alpine meadow. Journal of Plant Ecology 7(3), 231-239. doi: 10.1093/jpe/rtt030.

Zhou, X. (2019). The Reproductive Characteristics of Solidago canadensis under Simulated Climate Warming and Atmospheric Nitrogen Deposition. Master

Zhou, Z.X., Zhang, L.W., Liu, Y.Z., Zhang, K.P., Wang, W.R., Zhu, J.K., et al. (2022). Contrasting Effects of Nitrogen Addition on Vegetative Phenology in Dry and Wet Years in a Temperate Steppe on the Mongolian Plateau. Frontiers in Plant Science 13. doi: 10.3389/fpls.2022.861794.

# 2 Supplementary Figures and Tables

## 2.1 Supplementary Table 1. A list of species included in the article.

|  | Family | Species |
| --- | --- | --- |
| 1 | Amaranthaceae Juss. | Kochia prostrata |
| 2 | Amaranthaceae Juss. | Allium bidentatum |
| 3 | Amaranthaceae Juss. | Allium mongolicum Regel |
| 4 | Amaranthaceae Juss. | Allium sikkimense |
| 5 | Amaranthaceae Juss. | Allium tenuissimu |
| 6 | Apiaceae Lindl. | Solidago canadensis L. |
| 7 | Asteraceae Bercht. & J. Presl | Solidago canadensis |
| 8 | Asteraceae Bercht. & J. Presl | Gnaphalium supinum |
| 9 | Asteraceae Bercht. & J. Presl | Helianthus annuus L |
| 10 | Asteraceae Bercht. & J. Presl | Heteropappus altaicus |
| 11 | Asteraceae Bercht. & J. Presl | Leontopodium nanum |
| 12 | Asteraceae Bercht. & J. Presl | Ligularia virgaurea |
| 13 | Asteraceae Bercht. & J. Presl | A. trullifolia var.linearis |
| 14 | Asteraceae Bercht. & J. Presl | Artemisia frigida |
| 15 | Asteraceae Bercht. & J. Presl | Artemisia frigida Willd |
| 16 | Asteraceae Bercht. & J. Presl | Saussurea hieracioides |
| 17 | Asteraceae Bercht. & J. Presl | Saussurea nigrescens |
| 18 | Asteraceae Bercht. & J. Presl | Saussurea pulchra Lipsch |
| 19 | Asteraceae Bercht. & J. Presl | Artemisia subulata |
| 20 | Asteraceae Bercht. & J. Presl | Chamaesium paradoxum H. Wolff |
| 21 | Betulaceae Gray | Betula platyphylla Sukaczev |
| 22 | Brassicaceae Burnett | Eruca sativa Mill. |

Continued Table 1

|  | Family | Species |
| --- | --- | --- |
| 23 | Brassicaceae Burnett | Brassica rapa |
| 24 | Combretaceae R. Br. | Terminalia sericea |
| 25 | Convolvulaceae Juss. | Convolvulus ammannii |
| 26 | Cyperaceae Juss. | Scirpus pumihus |
| 27 | Cyperaceae Juss. | Cyperus malaccensis subsp. monophyllus (Vahl) T. Koyama |
| 28 | Cyperaceae Juss. | Kobresia humilis |
| 29 | Cyperaceae Juss. | Kobresia pygmaea |
| 30 | Cyperaceae Juss. | Kobresia setchwanensis |
| 31 | Cyperaceae Juss. | Trichophorum distigmaticum |
| 32 | Dunaliellaceae | S. aliena |
| 33 | Euphorbiaceae | Euphorbia esula |
| 34 | Fagaceae Dumort. | Medicago sativa |
| 35 | Fagaceae Dumort. | Fagus longipetiolata Seem |
| 36 | Gentianaceae Juss. | Gentiana aristata |
| 37 | Gentianaceae Juss. | Gentiana crassicaulis |
| 38 | Gentianaceae Juss. | Halenia elliptica |
| 39 | Gentianaceae Juss. | Lomatogonium carinthiacum |
| 40 | Gentianaceae Juss. | Geranium pylzowianum |
| 41 | Gentianaceae Juss. | Carex scaposa |
| 42 | Gentianaceae Juss. | Erodium botrys |
| 43 | Gentianaceae Juss. | Geranium dissectum |
| 44 | Leguminosae | Astagahus polycladus |

Continued Table 1

|  | Family | Species |
| --- | --- | --- |
| 45 | Leguminosae | Gentiana sino-ornata |
| 46 | Leguminosae | Gentianopsis barbata |
| 47 | Leguminosae | Gentianopsis paludosa |
| 48 | Leguminosae | Oxytropis ochrocephala |
| 49 | Leguminosae | Tibetia himalaica |
| 50 | Leguminosae | Vicia sativa L. |
| 51 | Malvaceae Juss. | Sabz pari |
| 52 | Orobanchaceae Vent. | Pedicularis kansuensis |
| 53 | Parnassiaceae | Pamassia trinervis |
| 54 | Pedaliaceae R. Br. | Sesamum indicum L. |
| 55 | Pinaceae Spreng. | Larix principis-rupprechtii |
| 56 | Pinaceae Spreng. | Abies balsamea |
| 57 | Pinaceae Spreng. | Picea mariana |
| 58 | Pinaceae Spreng. | Pinus elliottii |
| 59 | Plantaginaceae Juss. | Veronica eriogyne |
| 60 | Plantaginaceae Juss. | Solidago canadensis L. |
| 61 | Poaceae Barnhart | Agropyron cristatum |
| 62 | Poaceae Barnhart | Agroslishugoniana |
| 63 | Poaceae Barnhart | Agroslistrinii |
| 64 | Poaceae Barnhart | Agrostis hugoniana |
| 65 | Poaceae Barnhart | Avena spp. |
| 66 | Poaceae Barnhart | Bromus diandrus |
| 67 | Poaceae Barnhart | Bromus hordeaceus |

Continued Table 1

|  | Family | Species |
| --- | --- | --- |
| 68 | Poaceae Barnhart | Cleistogenes songorica |
| 69 | Poaceae Barnhart | Elymus nutans Griseb. |
| 70 | Poaceae Barnhart | Elynsmians |
| 71 | Poaceae Barnhart | Fesluca ovina |
| 72 | Poaceae Barnhart | Festuca coelestis |
| 73 | Poaceae Barnhart | Kobresia capillifolia |
| 74 | Poaceae Barnhart | Koeleria cristata |
| 75 | Poaceae Barnhart | Leymus chinensis |
| 76 | Poaceae Barnhart | L. multiflorum |
| 77 | Poaceae Barnhart | Oryza sativaL |
| 78 | Poaceae Barnhart | P. tenuiflora |
| 79 | Poaceae Barnhart | Poa poophagorum |
| 80 | Poaceae Barnhart | Poa pratensis |
| 81 | Poaceae Barnhart | Roegneria nutans |
| 82 | Poaceae Barnhart | Stipa aliena |
| 83 | Poaceae Barnhart | Stipa breviflora |
| 84 | Poaceae Barnhart | Stipa capillata |
| 85 | Poaceae Barnhart | Stipa krylovii |
| 86 | Poaceae Barnhart | Vulpia myuros |
| 87 | Poaceae Barnhart | Triticum aestivum L. |
| 88 | Poaceae Barnhart | Sorghum bicolor |
| 89 | Poaceae Barnhart | Zea mays Continued Table S1 |
| 90 | Poaceae Barnhart | Oryza sativa L. |

Continued Table 1

|  | Family | Species |
| --- | --- | --- |
| 91 | Poaceae Barnhart | Hordeum vulgare |
| 92 | Poaceae Barnhart | Solidago canadensis L. |
| 93 | Polygonaceae Juss. | Rumex patientia |
| 94 | Ranunculaceae Juss. | Deiphinium kan |
| 95 | Ranunculaceae Juss. | Anemone obnusiloba |
| 96 | Ranunculaceae Juss. | Anemone rivularis |
| 97 | Ranunculaceae Juss. | Anemone trullifolia |
| 98 | Ranunculaceae Juss. | Delphinium kamaonense |
| 99 | Ranunculaceae Juss. | Ranunculus tanguticus |
| 100 | Ranunculaceae Juss. | Trollius farreri |
| 101 | Ranunculaceae Juss. | Thalictrum alpinum |
| 102 | Ranunculaceae Juss. | Anemone obtusiloba D. Don |
| 103 | Ranunculaceae Juss. | Thalictrum alpinum L. |
| 104 | Ranunculaceae Juss. | Anemone coelestina |
| 105 | Ranunculaceae Juss. | Anemone rivularis |
| 106 | Ranunculaceae Juss. | Veronica eriogyne H. Winkl. |
| 107 | Ranunculaceae Juss. | Elymus nutans Griseb. |
| 108 | Ranunculaceae Juss. | Poa albertii subsp. |
| 109 | Ranunculaceae Juss. | Delphinium kamaonense |
| 110 | Rosaceae Juss. | Polentilla.fiagarioides |
| 111 | Rosaceae Juss. | Potentilla acaulis |
| 112 | Rosaceae Juss. | Potentilla anserina |
| 113 | Rosaceae Juss. | Potentilla bifurca |

Continued Table 1

|  | Family | Species |
| --- | --- | --- |
| 114 | Rosaceae Juss. | Potentilla cuneata |
| 115 | Rosaceae Juss. | Potentilla fragarioides |
| 116 | Rosaceae Juss. | Potentilla saundersiana |
| 117 | Rosaceae Juss. | Potentilla tanacetifolia |
| 118 | Rosaceae Juss. | Potentilla fragarioides L. |
| 119 | Rosaceae Juss. | Solidago canadensis L. |
| 120 | Rubiaceae | Galium verum |
| 121 | Salicaceae Mirb. | Salix spp |
| 122 | Sapindaceae Juss. | Mauritius litchi |
| 123 | Sapindaceae Juss. | Aesculus chinensis Bunge |
| 124 | Scrophulariaceae | Euphrasia regelii |
| 125 | Umbelliferae | Tongoloa tenuiolia |

**Supplementary Table 2** Selected model weights

|  | model | aicc | weights |
| --- | --- | --- | --- |
| 1 | yi ~ 1 + temperature | -1598.470 | 0.4935595694 |
| 2 | yi ~ 1 + temperature + N | -1596.854 | 0.2199789947 |
| 3 | yi ~ 1 + temperature + precipitation | -1596.604 | 0.1940738511 |
| 4 | yi ~ 1 + temperature + precipitation + N | -1595.055 | 0.0894777055 |
| 5 | yi ~ 1 | -1585.761 | 0.0008581630 |
| 6 | yi ~ 1 + precipitation | -1585.636 | 0.0008062416 |
| 7 | yi ~ 1 + precipitation + N | -1585.200 | 0.0006482739 |
| 8 | yi ~ 1 + N | -1585.036 | 0.0005972007 |

**Supplementary Table 3** Optimal model set

|  | model | | aicc | weights |
| --- | --- | --- | --- | --- |
| 1 | yi ~ 1 + temperature | -1598.470 | | 0.4935596 |
| 2 | yi ~ 1 + temperature + N | -1596.854 | | 0.2199790 |

## Supplementary Figure 1. Weights of each influencing factor in the optimal model, With importance >0.8 as the critical value, >0.8 indicates that the factor is important


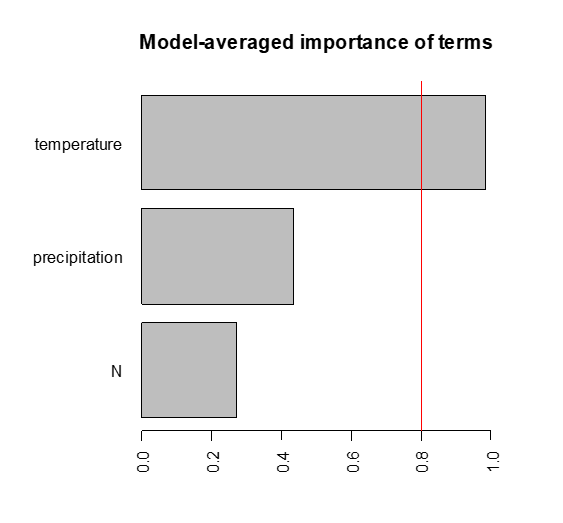


**Supplementary Figure 2** Preference analysis, The funnel shape is symmetrical, and the results are less affected by publication preference.


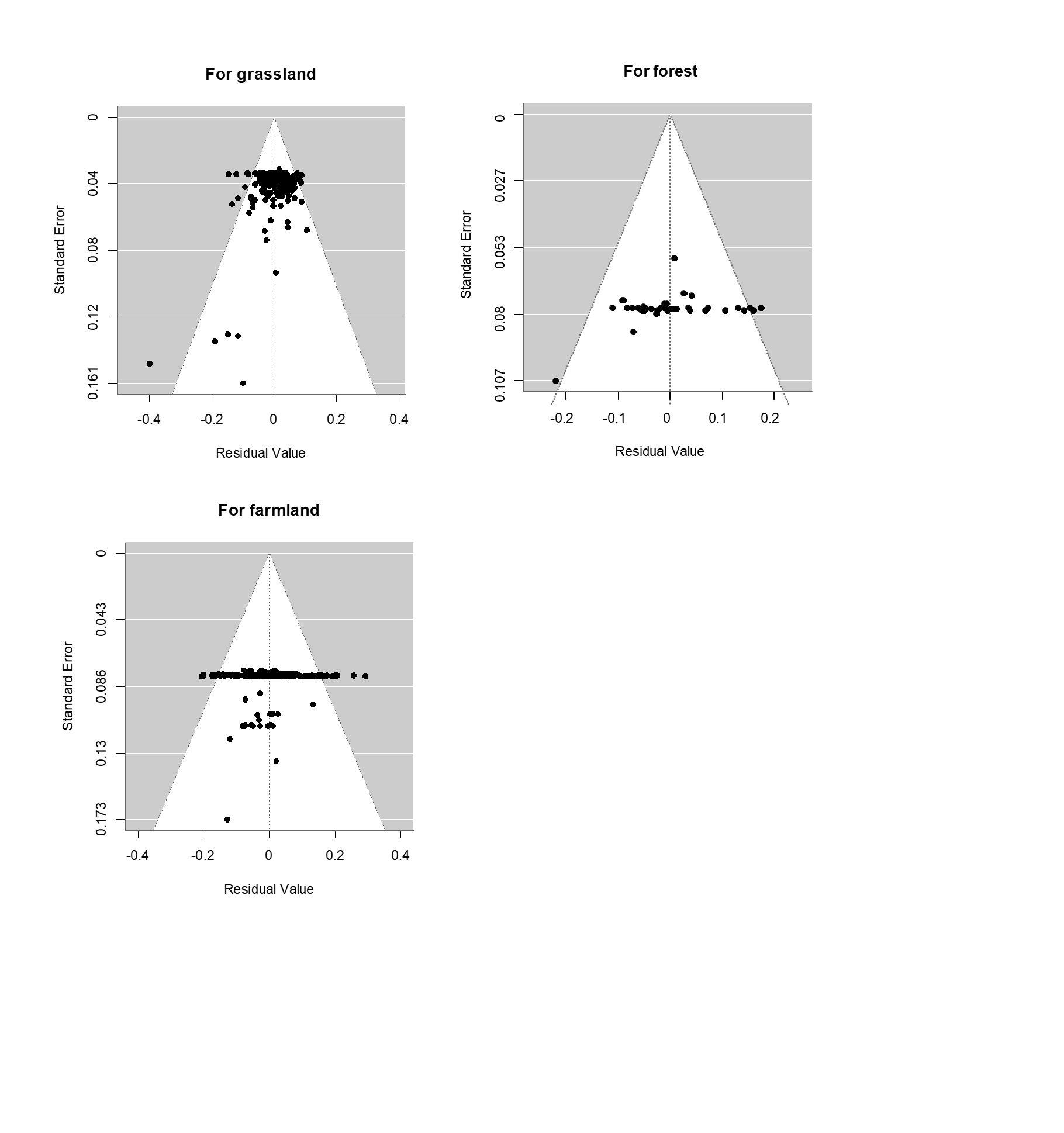


**Supplementary Figure3.** Qq normal diagram, The normality test is to check whether the residual of the model matches, All the points fall into the confidence interval, indicating that the model fits well.

**
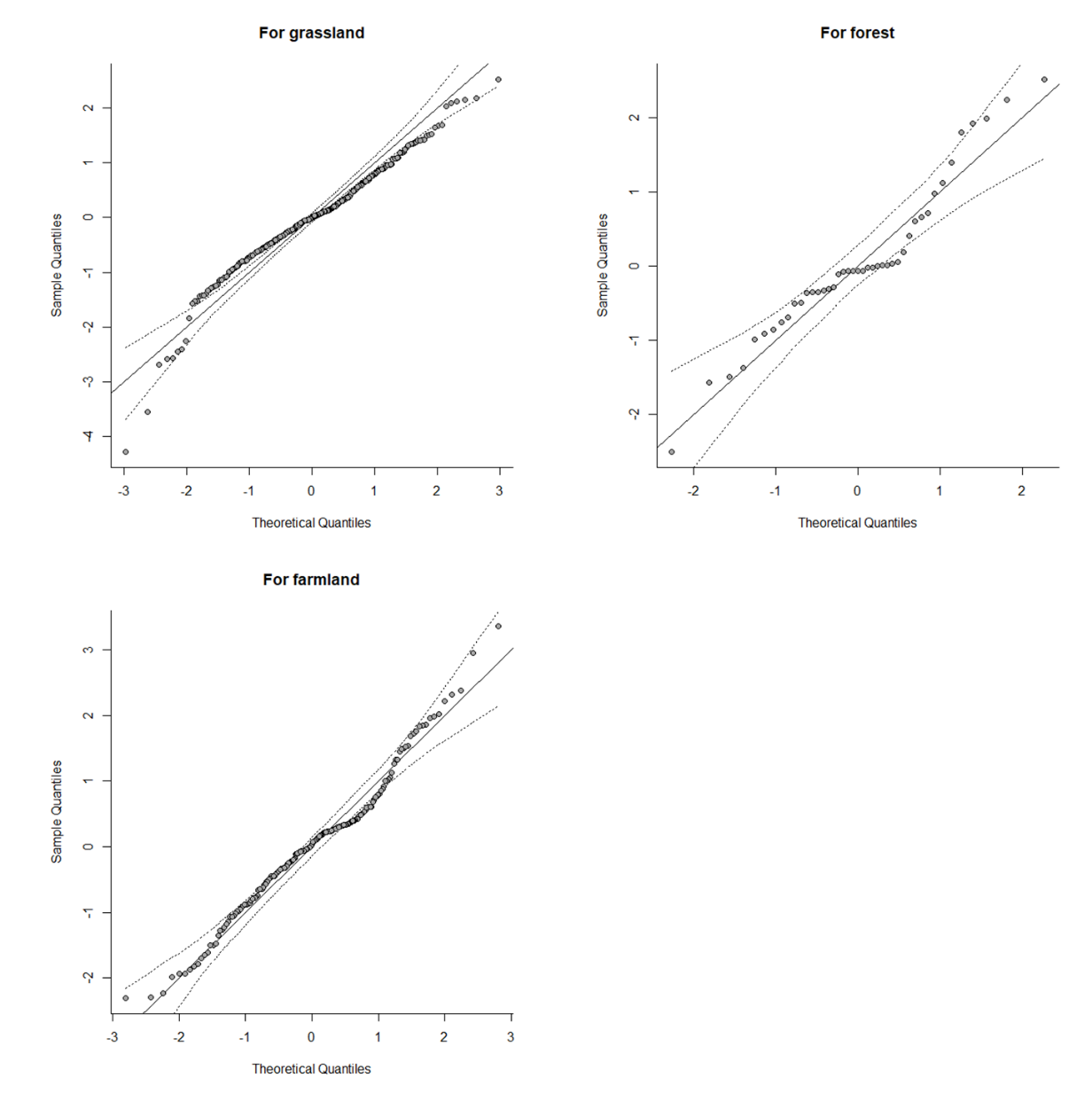
**
